# Independent Component Analysis Identifies the Modulons Expanding the Transcriptional Regulatory Networks of Enterohemorrhagic *Escherichia coli*

**DOI:** 10.3389/fmicb.2022.953404

**Published:** 2022-06-24

**Authors:** Hanhyeok Im, Ju-Hoon Lee, Sang Ho Choi

**Affiliations:** ^1^National Research Laboratory of Molecular Microbiology and Toxicology, Department of Agricultural Biotechnology, Seoul National University, Seoul, South Korea; ^2^Department of Agricultural Biotechnology, Center for Food and Bioconvergence, Seoul National University, Seoul, South Korea; ^3^Department of Agricultural Biotechnology, Research Institute of Agriculture and Life Science, Seoul National University, Seoul, South Korea

**Keywords:** EHEC, transcriptional regulatory network, machine learning, independent component analysis, transcriptome

## Abstract

The elucidation of the transcriptional regulatory networks (TRNs) of enterohemorrhagic *Escherichia coli* (EHEC) is critical to understand its pathogenesis and survival in the host. However, the analyses of current TRNs are still limited to comprehensively understand their target genes generally co-regulated under various conditions regardless of the genetic backgrounds. In this study, independent component analysis (ICA), a machine learning-based decomposition method, was used to decompose the large-scale transcriptome data of EHEC into the modulons, which contain the target genes of several TRNs. The locus of enterocyte effacement (LEE) and the Shiga toxin (Stx) modulons mainly consisted of the Ler regulon and the Stx prophage genes, respectively, confirming that ICA properly grouped the co-regulated major virulence genes of EHEC. Further investigation revealed that the LEE modulon contained the hypothetical Z0395 gene as a novel member of the Ler regulon, and the Stx modulon contained the *thi* and *cus* locus genes in addition to the Stx prophage genes. Correspondingly, the Stx prophage genes were also regulated by thiamine and copper ions known to control the *thi* and *cus* locus genes, respectively. The modulons effectively clustered the genes co-regulated regardless of the growth conditions and the genetic backgrounds of EHEC. The changed activities of the individual modulons successfully explained the differential expressions of the virulence and survival genes during the course of infection in bovines. Altogether, these results suggested that ICA of the large-scale transcriptome data can expand and enhance the current understanding of the TRNs of EHEC.

## Introduction

Transcriptional regulatory networks (TRNs) regulate the expression of the target genes for the pathogens to adapt to various environments. The understanding of TRNs and their target genes enables the prediction of molecular mechanisms by which pathogens cause disease and survive under host-specific conditions (Karmali, 2017). Advances in next-generation sequencing technologies facilitate analyzing the large-scale RNA-Seq and comparing the transcriptome of the pathogens grown under specific conditions or lacking a particular transcription factor(s) (TF) (Westermann et al., 2012; DuPai et al., 2020). However, the transcriptome data obtained from the genes expressed under specific experimental conditions or by a certain TF are still limited to comprehensively understand the TRNs and their target genes (Sastry et al., 2019; DuPai et al., 2020). Therefore, to overcome this limitation, studies have been performed to analyze bioinformatically the large-scale transcriptome data of the pathogens and to define the modulons, the independent sets of genes co-regulated under various conditions regardless of their genetic backgrounds (Saelens et al., 2018; Sastry et al., 2019; DuPai et al., 2020; Tan et al., 2020).

Enterohemorrhagic *Escherichia coli* (EHEC) causes a broad spectrum of human illnesses ranging from mild diarrhea to hemolytic uremic syndrome, often leaving permanent damage to the kidney (Karmali, 2017). The TRNs of Ler and Shiga toxin (Stx) prophage encoding the major virulence factors of EHEC have been studied extensively to understand the molecular pathogenesis of the pathogen. Ler, encoded by *ler*, regulates the locus of enterocyte effacement (LEE) genes necessary to form attaching and effacing (AE) lesions, the central pathogenesis of EHEC (Kenny et al., 1997; Mellies et al., 1999; Elliott et al., 2000; Tobe et al., 2006). Ler also regulates the genes encoding non-LEE-encoded effector (Nle) proteins crucial for forming AE lesions (Kelly et al., 2006; Li et al., 2006; Tobe et al., 2006), demonstrating that the Ler TRN contains additional non-LEE genes. Additionally, the Stx prophage TRNs include *stx1* and *stx2* of EHEC, located in the CP-933V and BP-933W prophages, respectively (Xu et al., 2012). The expressions of the Stx genes are regulated by the antiterminator *Qs* which allows the transcription of the prophage genes by preventing the formation of intrinsic terminators in their promoters (Casjens and Hendrix, 2015; Sy et al., 2020).

Meanwhile, TRNs also coordinate the expressions of the target genes for pathogens to survive under various growth conditions by recognizing the changes in the environmental signals. For example, the copper transport TRNs of EHEC consisting of *cusCFBA* involved in the detoxification of toxic heavy metals are induced by the high copper ions (Delmar et al., 2015). Other genes induced by environmental signals are generally suppressed by the global regulator H-NS encoded by *hns* (Atlung and Ingmer, 1997; Lang et al., 2007). Conversely, the target genes of certain TRNs also could be suppressed by the environmental signals. For example, The LEE genes of the Ler TRN are suppressed in the presence of indole, synthesized by the tryptophanase encoded by *tna* (Kumar and Sperandio, 2019). Similarly, the TRNs containing *thiBP* and *thiCEFGH* involved in the thiamine transport and biosynthesis, respectively, are also suppressed in the presence of thiamine (Vander Horn et al., 1993; Webb et al., 1998; Miranda-Rios et al., 2001).

In this study, independent component analysis (ICA), a machine learning method that decomposes a mixture of components into independent components (James and Hesse, 2005; Sastry et al., 2019; Tan et al., 2020), was used to decompose the large-scale transcriptome data of EHEC into the sets of independent modulon, which contains the target genes of several TRNs. The LEE and the Stx modulons mainly consisted of the target genes of the Ler and the Stx prophage TRNs, respectively, indicating that ICA properly grouped the sets of the co-regulated genes of EHEC into the modulons. Further investigation identified the Z0395 gene and the *thi* and *cus* locus genes as novel element genes of the LEE and Stx modulons, respectively. Accordingly, the Stx prophage genes were regulated by thiamine and copper ions known to control the *thi* and *cus* locus genes, respectively. Changed expressions of the modulons consisting of the inherently co-regulated genes also successfully explained the differential expressions of the virulence and survival genes of EHEC during the course of infection in bovines.

## Materials and Methods

### Generation of the Trimmed Transcriptome Data of Enterohemorrhagic *Escherichia coli*

The raw-sequencing reads of available RNA-Seq data of EHEC were retrieved from the Sequence Read Archive (SRA) database at the National Center for Biotechnology Information (NCBI)^1^ (see Supplementary Data Sheet 1). The reads were mapped to the reference genome of EHEC EDL933 (AE005174.2) using Spliced Transcripts Alignment to a Reference (STAR) (Dobin et al., 2013). The reads aligned to the reference genome were counted using the HTSeq (Anders et al., 2015). The genes with under ten fragments per million-mapped reads across the whole RNA-Seq data were removed before further analyses to ensure the quality of the data. The raw read counts were normalized using the trimmed mean of *M* values (TMM) method from the R *edgeR* package (Robinson et al., 2010; Robinson and Oshlack, 2010). The normalized data with *R*^2^ < 0.9 between biological replicates were discarded to trim the technical noise (Supplementary Figure 1A). The trimmed transcriptome data were log-transformed (log_2_ TMM + 1) for further analysis (see Supplementary Data Sheet 2).

### Identification of the Modulons by Using Independent Component Analysis

Independent component analysis was conducted to the trimmed transcriptome data as previously described (Sastry et al., 2019). Firstly, the trimmed transcriptome data are centered by using the mean read counts of the transcriptome data of the EHEC EDL933 grown in M9 minimal medium. Then, ICA from the Scikit-learn Python package (Varoquaux et al., 2015), based on the hyperparameters of convergence tolerance of 10^–8^ and component number of 88 (the size of transcriptome data), was performed on the centered transcriptome data to construct the independent gene components. ICA was executed 256 times with random seeds, and the resulting independent gene components were clustered by using density-based spatial clustering of applications with noise (DBSCAN) to identify robust independent gene components (Ester et al., 1996). DBSCAN from the Scikit-learn Python package was conducted based on the hyperparameters of epsilon of 0.1 and minimum cluster seed size of 128 (50% of the execution times of ICA). In order to select the co-regulated genes of the robust independent components, the D’Agostino *K*^2^ test, which measures the skewness and kurtosis of distribution, was performed on the gene coefficients of the element genes in the independent components (D’Agostino et al., 1990). The element gene with the greatest absolute coefficient in each independent gene component was repeatedly removed, and the D’Agostino *K*^2^ test statistic was calculated for each removal. If the test statistic dropped below a cut-off, the removed genes were defined as the co-regulated genes of the independent component.

To determine the *K*^2^ test statistic cut-off, a two-sided Fisher’s exact test was performed between the previously known regulons of the *E. coli* regulators and the top 25 element genes of the independent gene components (Gama-Castro et al., 2011; Fang et al., 2017; Sastry et al., 2019). Among the regulators, the regulator with the lowest *P*-value was linked to each independent gene component. Then, the F1 scores were calculated between the regulons of the component-linked regulators and the co-regulated genes of the independent gene components selected based on the *K*^2^ test statistic cut-off varying from 1,500 to 2,500. Because the average of calculated F1 scores showed a maximum value at the *K*^2^ test statistic cut-off of 1,800 (Supplementary Figure 1D), the statistic cut-off was used to define the modulons. The independent components with less than 5 co-regulated genes were discarded, and thus the 64 modulons were identified from the 85 independent components. The 64 modulons were named after their related regulator or biological function (e.g., H-NS or LEE). Detailed information of the modulons, such as the related TF or biological function, the co-regulated genes, and the gene coefficients, was available in Supplementary Data Sheet 3.

### Calculation of Cumulative Explained Variance for Principal Component Analysis and Independent Component Analysis

The principal component analysis (PCA) of the trimmed transcriptome data was performed with the Scikit-learn Python package (Varoquaux et al., 2015). The cumulative explained variance (CEV) for the PCA results was calculated by sequentially adding the explained variance ratios of the principal components using the Scikit-learn and NumPy Python packages (Varoquaux et al., 2015; Harris et al., 2020). The CEV for the ICA results was calculated as previously described in EEGLAB (Delorme and Makeig, 2004). The Matplotlib Python package was used to visualize the CEV for the PCA and ICA results (Hunter, 2007).

### The Correlation Analyses of the Expression Levels of the Genes or the Activities of the Modulons

The expression levels of the genes and the activities of the modulons were obtained from Supplementary Data Sheet 2, 4, respectively. The Pearson correlation analyses between the expression levels of the genes and the activities of the modulons were performed with the SciPy Python package (Virtanen et al., 2020). The Pearson correlations between the expressions of the different genes were performed with the Pandas Python package (McKinney, 2010). The Matplotlib Python package was used to visualize the plots of the correlation analyses (Hunter, 2007).

### Searching for the Ler Binding Site of the Z0395 Gene

The binding motif of Ler was discovered from the specific binding sequences of Ler, which were previously reported by Abe et al. (2008), by using the Multiple Expectation maximizations for Motif Elicitation (MEME) (Bailey et al., 2006). The Ler binding site was predicted *in silico* by searching the upstream sequences of the Z0395 gene by using the Find Individual Motif Occurrences (FIMO) (Grant et al., 2011).

### Strains, Plasmids, and Culture Conditions

All the strains and plasmids used in this study are listed in Supplementary Table 1. Unless otherwise noted, the *E. coli* strains were grown aerobically in Luria-Bertani (LB) medium at 37°C. *E. coli* DH5α was used as a cloning host, and EHEC EDL933 was used as the wild-type (WT). The pCas and pTargetF plasmids required for mutant construction of *E. coli* were obtained from Addgene (plasmid #62225 and #62226) (Jiang et al., 2015).

### Generation of a *ler* Deletion Mutant

The *ler* (Z5140) was inactivated by deletion (207 of 390 bp) of the coding region using the clustered regularly interspaced short palindromic repeats (CRISPR)-Cas9 system as previously described (Jiang et al., 2015). Briefly, two amplicons designed to carry homologous arms with 5′- and 3′-flanking regions of *ler* were amplified by PCR using LER-F1-F and -R or LER-F2-F and -R pairs of primers (Supplementary Table 2). Both amplicons were fused into donor DNA by overlap extension PCR using the primer pairs of LER-F1-F and LER-F2-R. Replacing the N_20_ of pTargetF to target *ler* was performed using the Site-Directed Mutagenesis Kit (NEB, Beverly, MA, United States) according to the manufacturer’s protocols. The N_20_ replaced pTargetF targeting *ler* was designated as pTargetF-ler (Supplementary Table 1). The EDL933 electrocompetent cells harboring pCas were prepared as previously described (Sharan et al., 2009). For genome editing, 400 ng of donor DNA and 100 ng of pTargetF-ler were co-electroporated into the EDL933 electrocompetent cells. The construction of the *ler* deletion mutant was confirmed by PCR.

### Quantitative Reverse Transcription-PCR

The total RNA of the EDL933 strains grown under various conditions were isolated to determine the relative transcript levels of genes of interest by quantitative reverse transcription-PCR (qRT-PCR). In detail, to determine the relative transcript levels of the Z0395 gene, the EHEC strains were grown in low-glucose Dulbecco’s modified Eagle’s medium (Merck, Darmstadt, Germany) at 37°C to an *A*_600_ of 1.0. To determine the relative transcript levels of *thiB*, *thiC*, and *stx2a*, the EHEC strains were grown in M9 minimal medium with or without thiamine at 37°C to an *A*_600_ of 0.75. Finally, to determine the relative transcript levels of *cusC* and *stx2a*, the EHEC strains were grown in LB medium with different levels of CuSO_4_ at 37°C to an *A*_600_ of 1.0. The total RNAs of the strains were isolated using the RNeasy mini kit (Qiagen, Valencia, CA, United States). For qRT-PCR, the concentrations of the total RNAs were measured by using a NanoDrop One spectrophotometer (Thermo Scientific, Waltham, MA, United States), and cDNA was synthesized from 100 ng of total RNA by using iScript cDNA synthesis kit (Bio-Rad, Hercules, CA, United States). Real-time PCR amplification of the cDNA was performed by using CFX96 real-time PCR detection system (Bio-Rad) with specific primer pairs (Supplementary Table 2) as described previously (Jang et al., 2017). The relative transcript levels of the genes were calculated by using the transcript levels of the glyceraldehyde-3-phosphate dehydrogenase (GAPDH) as the internal reference for normalization (Kijewski et al., 2020).

## Results

### The Modulons Containing the Target Genes of Several Transcriptional Regulatory Networks of Enterohemorrhagic *Escherichia coli* Are Identified by Using Independent Component Analysis

Independent component analysis, a machine learning-based decomposition method, was used to decompose the large-scale transcriptome data of EHEC into the modulons containing the target genes of several TRNs. For this purpose, the trimmed 88 transcriptome data of EHEC (*R*^2^ ≥ 0.9 between biological replicates) (Supplementary Figure 1A and Supplementary Data Sheet 1, 2) were decomposed into the 85 independent gene components (Supplementary Data Sheet 3). ICA was also used to calculate the overall expression levels of the decomposed 85 components: the activities of the components in a specific condition. The activities of the 85 independent components (Supplementary Data Sheet 4) successfully explained 83% of the total expression variance of the 88 transcriptome data (Supplementary Figure 1B), validating that ICA properly decomposed the transcriptome data of EHEC into the independent gene components.

The 85 independent components contain the element genes with varied gene coefficients that represent the degree of the regulatory effect on the expressions of the genes. The element genes with a positive or negative gene coefficient indicate that their expressions are proportionally or inversely regulated along with the activities of the independent component, respectively. Unless otherwise noted, gene coefficient signs of element genes in an independent component are positive. Most of the gene coefficients of element genes in an independent component were distributed close to 0 (Supplementary Figure 1C), indicating that the expressions of only a few element genes significantly rely on the activities of an independent component. The distribution of the gene coefficients was reexamined by the statistical analysis, D’Agostino *K*^2^ test (D’Agostino et al., 1990; Sastry et al., 2019), to select only the genes with the coefficients far away from 0. As a result, the element genes with the gene coefficients over a cut-off, D’Agostino *K*^2^ test statistic 1,800 (Supplementary Figure 1D), were selected as the co-regulated genes of an independent component and defined as the modulons (see section “Materials and Methods” for details on the selection process). Consequently, a total of 64 modulons were identified from the 85 independent components. The 64 modulons with detailed information are presented in Supplementary Data Sheet 3.

### The Locus of Enterocyte Effacement and the Shiga Toxin Modulons Contain the Ler Regulon and the Shiga Toxin Prophage Genes, Respectively

**FIGURE 1:**
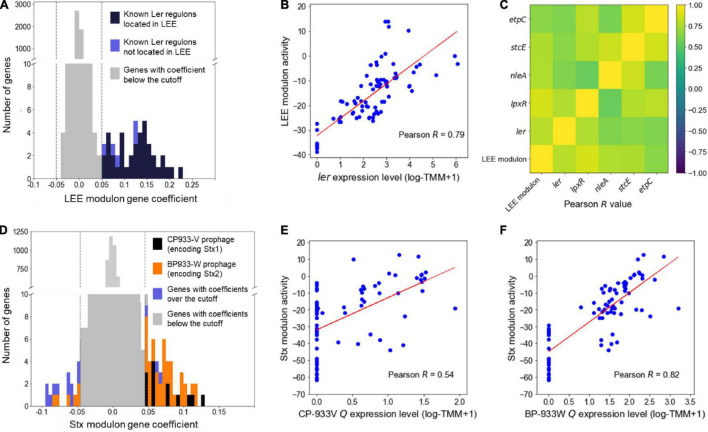
Validation of the LEE and Stx modulons. Histograms of the gene coefficients of the element genes in the LEE **(A)** and the Stx modulons **(D)**. The dotted lines in the boxes show the cut-off values of the gene coefficients in each modulon. The colors of the bars represent the classifications of the genes as indicated in the plots. The scatter plots of the activities of the LEE modulon and the expression levels of the *ler* **(B)**, the activities of the Stx modulon and the expression levels of the CP-933V antiterminator *Q* **(E)**, and the activities of the Stx modulon and the expression levels of the BP-933W antiterminator *Q* **(F)**. The Pearson *R* values between the activities of the modulons and the expression levels of their related TF are denoted in the boxes. Each dot of the plots represents a single biological replicate. Red lines represent the regression lines of the plots. **(C)** Ordered correlation matrix. Colors indicate the Pearson *R* values between the activities of the LEE modulon and the expression levels of the Ler regulon that are not located in the LEE. Yellow and indigo represent the strongest positive (+1) and negative (–1) correlation, respectively.

The modulons mainly consisting of the LEE and the Stx prophage genes encoding the major virulence factors of EHEC were defined as the LEE and the Stx modulon, respectively, and were further investigated to confirm whether the modulons adequately contain the sets of co-regulated genes. The LEE modulon contains 44 genes, of which 39 were the LEE genes (Figure 1A). Also, the activities of the LEE modulon were strongly correlated with the expression levels of *ler* (Pearson *R* = 0.79, *P* < 10^–10^) (Figure 1B), indicating that the LEE modulon primarily consisted of the Ler regulon. Furthermore, the LEE modulon contained *lpxR*, *nleA*, *stcE*, and *etpC*, which were not located in the LEE but known as the Ler regulon (Figure 1A; Grys et al., 2005; Tobe et al., 2006; Roe et al., 2007; Ogawa et al., 2018). The activities of the LEE modulon were also highly correlated with the expression levels of these genes (Pearson *R* > 0.5, *P* < 10^–5^) (Figure 1C), indicating that the modulon properly contained the genes located separately but co-regulated by Ler. Similarly, the Stx modulon contained the CP-933V and BP-933W prophage genes that include *stx1* and *stx2*, respectively (Figure 1D). The activities of the Stx modulon were also highly correlated with the expression levels of the antiterminator *Qs* (Pearson *R* > 0.5, *P* < 10^–5^) (Figures 1E,F), indicating that the Stx modulons mainly consisted of the Stx prophage genes co-related by the antiterminator *Qs*. Consequently, these results validated that the modulons, the independent sets of co-regulated genes, were appropriately identified from the large-scale transcriptome data by using ICA.

### The Locus of Enterocyte Effacement Modulon Contains the Z0395 Gene as a Novel Member of the Ler Regulon

**FIGURE 2:**
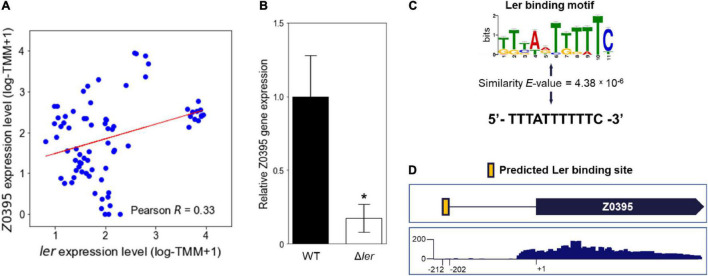
The Z0395 gene is a member of the Ler regulon. **(A)** The scatter plot of the expression levels of the Z0395 gene and *ler*. Each dot of the plot represents a single biological replicate. Red line represents the regression line of the plot. The Pearson *R* value between the expression levels of the Z0395 gene and *ler* is denoted in the box. **(B)** The relative expression levels of the Z0395 gene in the WT and *ler* deletion mutant. The levels of the Z0395 gene transcripts were determined by qRT-PCR, and the Z0395 gene transcript levels in the WT were set to 1. Error bars represent the SD from four independent experiments. Statistical significance was determined by the Student’s *t*-test (*P < 0.05). WT, EDL933; Δ*ler*, *ler* deletion mutant. **(C)** The Ler binding motif depicted in the logo and the Ler binding sequence predicted *in silico* found at the Z0395 gene upstream region. The height of the letters in the logo represents the information contents of the position in bits. The similarity between the Ler binding motif (top) and the predicted binding sequence (bottom) are denoted as *E*-value. **(D)** Location of the Ler binding sequence *in silico* predicted in the Z0395 gene upstream region. The Ler binding sequence is located from –212 to –202 region of the Z0395 ORF, represented as a yellow box. The bellow box represents the coverage plot of the reads mapped to the Z0395 gene. The transcriptome data of EHEC EDL933 grown in M9 minimal medium were used to generate the plot. The *y*-axis represents the normalized number of reads per base. The average number of reads of the biological triplicates are shown in the plot.

The element genes of the LEE modulon were further investigated to analyze the target genes of the Ler TRN encoding the major virulence factor of EHEC. The LEE modulon included a hypothetical gene, the Z0395 gene, which is not located in the LEE (Z5099-5141) and is not known as the Ler regulon. Since most of the genes in the LEE modulon were the Ler regulon (Figure 1A), it was possible that the Z0395 gene is also a member of the Ler regulon. To examine the possibility, the relationship between the expressions of the Z0395 gene and *ler* was analyzed. As shown in Figure 2A, the expressions of the Z0395 gene and *ler* were positively correlated (Pearson *R* = 0.33, *P* < 0.05). Thus, to further verify the effect of Ler on the expression of the Z0395 gene, the transcript levels of the Z0395 gene in the WT and the *ler* deletion mutant (Δ*ler*) were compared. The transcript level of the Z0395 gene was greatly reduced in Δ*ler* (Figure 2B), confirming that Ler activates the Z0395 gene expression at the transcription level. To examine whether Ler directly binds to the probable promoter region of the Z0395 gene, the upstream region of the Z0395 gene was scanned *in silico* with the binding motif of Ler. The motif-based sequence analysis predicted one Ler binding sequence located in the −212 to −201 region from the open reading frame (ORF) of the Z0395 gene (*P* < 10^–5^) (Figures 2C,D). Taken together, these results indicated that Ler regulates the expression of the Z0395 gene by directly binding to its upstream region, supporting that the Z0395 gene in the LEE modulon is a novel member of the Ler regulon.

### The Shiga Toxin Modulon Contains the *thi* and *cus* Locus Genes in Addition to the Shiga Toxin Prophages

**FIGURE 3:**
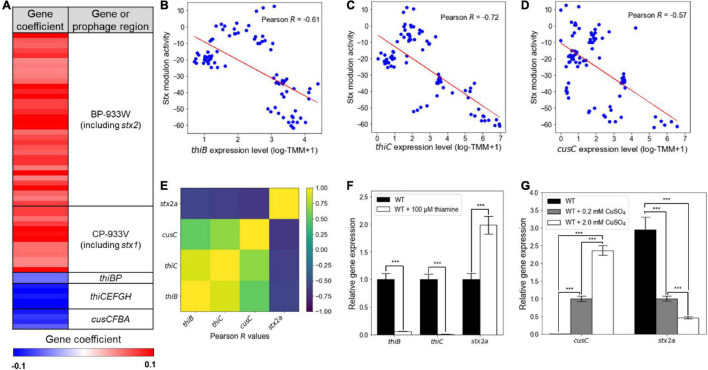
The contrary expression patterns of the *thi* and *cus* locus genes to those of the Stx prophage genes. **(A)** Heatmap for the gene coefficients in the Stx modulon. Red and blue represent the high (+0.1) and low (–0.1) gene coefficient, respectively. The scatter plots of the activities of the Stx modulon and the expression levels of *thiB* **(B)**, *thiC* **(C)**, and *cusC* **(D)**. Each dot of the plots represents a single biological replicate. Red lines represent the regression lines of the plots. The Pearson *R* values between the activities of the Stx modulon and the expression levels of each gene of the plot are denoted in the boxes. **(E)** Ordered correlation matrix. Colors indicate the Pearson *R* values between the expression levels of *thiB*, *thiC*, *cusC*, and *stx2a*, as indicated. Yellow and indigo represent the strongest positive (+1) and negative (–1) correlation, respectively. **(F,G)** The relative expression levels of genes of interest in the WT grown under the different levels of thiamine and copper ions. The transcript levels of *thiB*, *thiC*, and *stx2a* in the WT with or without thiamine were determined by qRT-PCR, and the transcript levels of each gene in the WT were set to 1 **(F)**. The transcript levels of *cusC* and *stx2a* in the WT with the different levels of CuSO_4_ were also determined by qRT-PCR, and the transcript levels of each gene in the WT with 0.2 mM CuSO_4_ were set to 1 **(G)**. Error bars represent the SD from four independent experiments. Statistical significance was determined by the Student’s *t*-test (***P < 10^–3^). WT, EDL933.

The element genes composing the Stx modulon were also further investigated. The Stx modulon contained the *thi* locus genes *thiBP* and *thiCEFGH* and the *cus* locus genes *cusCFBA*, which are not located in the Stx prophages (Figure 3A). These genes have negative gene coefficients in the Stx modulon, unlike the Stx prophage genes with positive gene coefficients (Figure 3A), indicating that the expressions of the *thi* and *cus* locus genes decrease as the activities of the Stx modulon increase. In accordance with this, the expression levels of *thiB, thiC*, and *cusC* were negatively correlated with the activities of the Stx modulon, with Pearson *R* −0.57 (*P* < 10^–5^), −0.72 (*P* < 10^–10^), and −0.61 (*P* < 10^–5^), respectively (Figures 3B–D). The negative relationship was further verified by the correlation analyses between the expression levels of *thiB, thiC*, and *cusC*, and those of *stx2a* (Pearson *R* < −0.5, P < 10^–8^) (Figure 3E), indicating that the expression patterns of the *thi* and *cus* locus genes were contrary to those of the Stx prophage genes. Since the expressions of the *thi* and *cus* locus genes are regulated by the levels of thiamine and copper ions, respectively (Vander Horn et al., 1993; Webb et al., 1998; Miranda-Rios et al., 2001; Delmar et al., 2015), the effect of the nutrients on the expression of *stx2a* was examined. Interestingly, the presence of thiamine significantly decreased the transcription of *thiB* and *thiC*, but increased that of *stx2a* (Figure 3F). Copper ions also increased the transcription of *cusC*, but decreased that of *stx2a* in a dose-dependent manner (Figure 3G). Consequently, the combined results revealed that the Stx modulon includes the *thi* and *cus* locus genes in addition to the Stx prophage genes, which are regulated by the levels of thiamine and copper ions.

### The Modulons Enhance the Clustering of the Genes Co-regulated Regardless of the Growth Conditions

**FIGURE 4:**
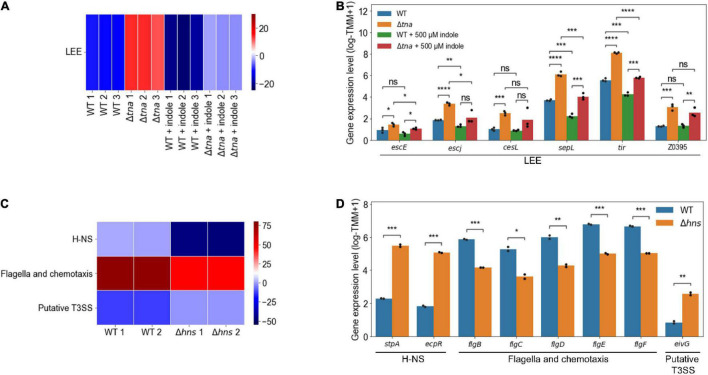
The changed activities of the modulons obtained from the transcriptome data of EHEC EDL933 and its isogenic mutants. Heatmap for the changed activities of the modulons obtained from the transcriptome data of the WT and Δ*tna* in the presence or absence of 500 μM indole **(A)**, and the WT and Δ*hns* **(C)**. The numbers on the bottom labels indicate a distinct single biological replicate. Red and blue represent the high and low activity of the modulon, respectively. WT, EDL933; Δ*tna, tna* deletion mutant; Δ*hns, hns* deletion mutant. The bar plots for the expression levels of the element genes of the modulons obtained from the transcriptome data of the WT and Δ*tna* in the presence or absence of 500 μM indole **(B)**, and the WT and Δ*hns* **(D)**. The modulon names of the element genes are denoted below the plots. The distinct colors of the bars represent the strains and the experimental conditions as indicated in the plots. Each dot on the bars represents a single biological replicate. Statistical significance was determined by the Student’s *t*-test (ns, not significant; *P < 0.05; **P < 10^–2^; ***P < 10^–3^; ****P < 10^–4^).

The element genes of the modulons are expected to be co-regulated under the various growth conditions. To verify this, it was investigated whether the expressions of the element genes in a modulon are altered together. The activities of the modulons were obtained from the transcriptome data of EHEC under different experimental conditions (Supplementary Figure 2). Among them, the significantly changed activities of the LEE modulon were observed from the transcriptome data of the WT and *tna* deletion mutant (Δ*tna*) in the presence or absence of 500 μM indole. In Δ*tna* imitating EHEC grown without indole, the activities of the LEE modulon increased significantly (*P* < 10^–5^) (Figure 4A). Accordingly, the expressions of the LEE genes, such as *escE*, *escJ*, *cesL*, *sepL*, and *tir*, increased significantly (Figure 4B). The addition of 500 μM indole significantly decreased the activities of the LEE modulon (*P* < 10^–5^) (Figure 4A) and thereby decreased the expressions of the LEE genes (Figure 4B). Interestingly, the changed activities of the LEE modulon altered the expressions of the non-LEE located hypothetical gene Z0395, the novel element gene of the LEE modulon (Figure 2A), in addition to the LEE genes (Figure 4B). These results indicated that the LEE modulon, as an example of the EHEC modulons, indeed enhanced the clustering of the genes co-regulated regardless of the growth conditions.

### The Modulons Improve the Clustering of the Genes Co-regulated Regardless of the Genetic Backgrounds

Significantly changed activities of H-NS (*P* < 10^–2^), flagella and chemotaxis (*P* < 10^–2^), and putative type III secretion system (T3SS) modulons (*P* < 10^–2^) were also observed from the transcriptome data of the WT and *hns* deletion mutant (Δ*hns*) (Figure 4C). The deletion of *hns* significantly decreased the activities of the H-NS modulon (*P* < 10^–2^) (Figure 4C). Since *stpA* and *ecpR*, the element genes of the H-NS modulon, have negative gene coefficients (Supplementary Data Sheet 3), the expressions of the genes increased significantly along with the decreased activities of the modulon in Δ*hns* (Figure 4D). The deletion of *hns* significantly decreased the activities of the flagella and chemotaxis modulon (*P* < 10^–2^) (Figure 4C) and thereby decreased the expressions of the flagella component genes *flgBCDEF* (Figure 4D). The deletion of *hns* also significantly increased the activities of the putative T3SS modulon (*P* < 10^–2^) (Figure 4C) and thereby increased the expressions of *eivG*, the putative T3SS component gene (Figure 4D). The *stpA* and *ecpR*, flagella component genes, and putative T3SS component genes, known as the H-NS regulon (Lang et al., 2007; Martínez-Santos et al., 2012; Ueda et al., 2013; Wan et al., 2016), were separately classified into the H-NS, flagella and chemotaxis, and putative T3SS modulons, respectively. These results indicated that the modulons successfully clustered the inherently co-regulated genes of EHEC regardless of the genetic backgrounds.

### The Modulons Enhance Understanding of the Differential Expressions of the Enterohemorrhagic *Escherichia coli* Virulence and Survival Genes

**FIGURE 5:**
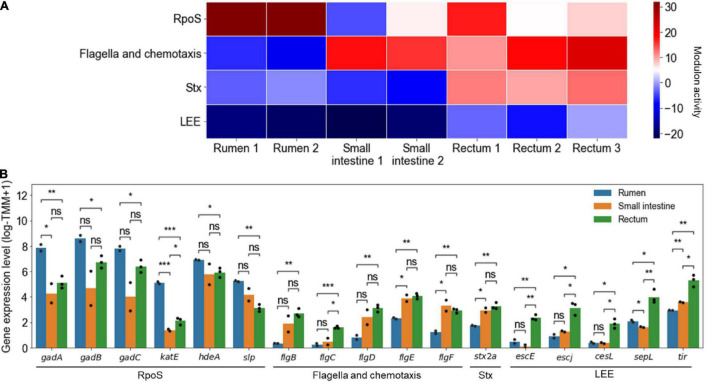
The changed activities of the modulons obtained from the transcriptome data of EHEC EDL933 in the different sites of the bovine GITs. **(A)** Heatmap for the changed activities of the modulons in the different sites of the bovine GITs. The numbers on the bottom labels indicate a distinct single biological replicate. Red and blue represent the high and low activity of the modulon, respectively. **(B)** The bar plots for the expression levels of the element genes of the modulons in the different sites of the bovine GITs. The modulon names of the element genes are denoted below the plot. The distinct colors of the bars represent the sites where EHEC was cultured as indicated in the plot. Each dot on the bars represents a single biological replicate. Statistical significance was determined by the Student’s *t*-test (ns, not significant; *P < 0.05; **P < 10^–2^; ***P < 10^–3^).

The changed activities of the modulons were analyzed from the transcriptome data previously obtained from EHEC in the different sites of the bovine GITs in order to confirm the differential gene expressions of the pathogen in the course of infection. For example, the activities of the RpoS, flagella and chemotaxis, Stx, and LEE modulons significantly changed in the different sites of the bovine GITs (Figure 5A). The activities of the RpoS modulon were significantly higher in the rumen than those in other sites of the bovine GITs (*P* < 10^–2^) (Figure 5A). Accordingly, the expressions of the element genes of the RpoS modulon, such as *gadABC* (Ling et al., 2008), *katE* (Schellhorn, 1995), *hdeA* (Dudin et al., 2013), and *slp* (Kabir et al., 2004), significantly increased in the rumen (Figure 5B). The activities of the flagella and chemotaxis modulon, and thereby the expressions of *flgBCDEF*, were significantly higher in the small intestine and rectum than in the rumen (*P* < 0.05) (Figures 5A,B). The activities of the Stx (*P* < 10^–3^) and LEE (*P* < 0.05) modulons, and thereby the expressions of the *stx2a*, *escE*, *escJ*, *cesL*, *sepL*, and *tir*, were significantly higher in the rectum than in other sites of the bovine GITs (Figures 5A,B). Consequently, these results indicated that the activities of the modulons could successfully explain the changed expressions of the virulence and survival genes in the different sites of the bovine GITs, enhancing understanding of the spatially differentiated gene expressions of EHEC during the course of infection.

## Discussion

In this study, ICA, a machine learning method that decomposes a mixture of components into independent components, was performed to decompose the large-scale transcriptome data of EHEC into the independent sets of co-regulated genes, the modulons. As a result, the trimmed 88 transcriptome data of EHEC (Supplementary Figure 1A) were decomposed into 64 independent modulons (Supplementary Figures 1B–D), which contain the target genes of the EHEC TRNs. The 64 modulons included the LEE and the Stx modulons mainly consisting of the LEE and the Stx prophage genes that encode the major virulence factors of EHEC, respectively (Figures 1A,D). The activities of the LEE modulon were strongly dependent on the expression level of *ler* (Figure 1B), and thus the LEE modulon mostly consisted of the Ler regulon. Moreover, the LEE modulon contained additional genes such as *lpxR*, *nleA*, *stcE*, and *etpC*, which are not located in the LEE but regulated by Ler (Figures 1A,C; Grys et al., 2005; Roe et al., 2007; Ogawa et al., 2018), indicating that ICA can precisely identify the LEE modulon to contain the target genes of the Ler TRN even not located in LEE. The Stx modulon contained the genes of the Stx prophages: CP-933V and BP-933W (Figure 1D). The activities of the Stx modulon were dependent on the expression levels of the antiterminator *Q*s (Figures 1E,F), indicating that the Stx modulon were adequately grouped with target genes of the Stx prophage TRNs. These results suggested that ICA successfully decomposed the large-scale transcriptome data of EHEC into the modulons.

The LEE modulon included a hypothetical Z0395 gene, which is not located within the LEE (Z5099-5141) and is not known as the Ler regulon. Interestingly, the expression of the Z0395 gene was predicted to increase along with the increased expression of *ler* (Figure 2A), suggesting that the Z0395 gene is a probable member of the Ler regulon. Experimentally, the deletion of *ler* significantly decreased the expression of the Z0395 gene (Figure 2B), confirming that the Z0395 gene in the LEE modulon is a new member of the Ler regulon. Furthermore, direct binding of Ler near the Z0395 gene was proposed by a previous ChIP-on-chip assay (Abe et al., 2008), and the Ler binding motif predicted *in silico* was found at the upstream region of the Z0395 gene (Figures 2C,D; Bailey et al., 2006; Grant et al., 2011). These results indicated that the Z0395 gene is a novel member of the Ler regulon, suggesting that the investigation of the modulons can discover new target genes of the current TRNs of EHEC.

The Stx modulon contained the non-prophage genes, the *thi* and *cus* locus genes, in addition to the Stx prophage genes (Figure 3A). The expression patterns of the *thi* and *cus* locus genes and those of other element genes in the Stx modulon were contrary (Figures 3B–D), and in detail, the expression levels of the *thiB*, *thiC*, and *cusC* genes have negative correlations with those of *stx2a* (Figure 3E). Interestingly, the levels of thiamine and copper ions known to control the expressions of the *thi* and *cus* locus genes, respectively (Vander Horn et al., 1993; Webb et al., 1998; Miranda-Rios et al., 2001; Delmar et al., 2015), inversely regulated the *stx2a* prophage gene (Figures 3F,G). Considering that thiamine is mostly produced by the gut microbiota (Said et al., 2001; Bhat and Kapila, 2017; Pan et al., 2017), the presence of thiamine could be an environmental signal for EHEC to suppress the *thi* locus genes and to induce the Stx virulence factors in the intestinal environments. Meanwhile, copper ions, mostly consumed with foods, are absorbed by the enterocytes in the upper small intestine and then left only in trace amounts in the large intestine (Doguer et al., 2018). Therefore, the relatively low copper ions also could be a signal for EHEC to suppress the *cus* locus genes and to induce the Stx virulence factors in the large intestine, the major colonization site for the pathogen (Vallance et al., 2002). Consequently, the investigation of the element genes of the Stx modulon could propose novel environmental signals such as the levels of thiamine and copper ions to control expressions of the Stx prophage genes, providing further understanding of the regulation of the TRNs of EHEC virulence factors.

The TRNs of bacteria primarily consist of the genes whose expressions are regulated together by a specific growth condition or the presence of a specific TF(s) (Sastry et al., 2019; DuPai et al., 2020). In contrast, the modulons of bacteria consist of the genes that are identified computationally and are expressed differentially together regardless of their growth conditions and the genetic backgrounds (Saelens et al., 2018; Sastry et al., 2019; Tan et al., 2020). Accordingly, novel gene Z0395, another element gene of the LEE modulon (Figure 2A), is expressed together with the LEE genes in the presence or absence of indole (Figures 4A,B). Additionally, the genes regulated by an identical TF can be classified into different modulons. For example, the flagella component genes and the putative T3SS component genes of the H-NS regulon were separately classified into the flagella and chemotaxis modulon, and putative T3SS modulon, respectively (Figures 4C,D). Altogether, these results indicated that the individual modulon successfully clustered a set of genes that are inherently co-regulated under the various conditions regardless of the genetic backgrounds of EHEC.

The changed activities of the modulons can be obtained from the transcriptome data of EHEC previously observed from the different sites of the bovine GITs. The activities of the RpoS modulon including the acid resistance genes, *gadABC*, increased significantly in the rumen, the acidic environment (Figures 5A,B; Ogawa et al., 2001; Ling et al., 2008; Chaucheyras-Durand et al., 2010). The activities of the flagella and chemotaxis modulon increased significantly in the small intestine and rectum (Figures 5A,B), which enables EHEC to move to more favorable niches (Naylor et al., 2003; Xu et al., 2012). The activities of the LEE and the Stx modulons increased significantly in the rectum (Figures 5A,B). The LEE genes encode the crucial adherence factors for colonizing the rectum, the primary colonization site of EHEC (Naylor et al., 2003). The Stxs also provide advantages for persistent colonization of EHEC by retarding the adaptive immune system at the bovine intestinal mucosa (Menge, 2020). Altogether, these results indicated that the changed activities of the modulons obtained from the transcriptome data could successfully explain the pathogenesis of EHEC during the course of infection in bovines.

## Conclusion

In summary, ICA of the large-scale transcriptome data identified the modulons consisting of the target genes of the EHEC TRNs. Further analysis of the modulons revealed that the Z0395 gene and the *thi* and *cus* locus genes are novel element genes of the LEE and Stx modulons, respectively. Concurrently, the Stx prophage genes were also regulated by thiamine and copper ions controlling the *thi* and *cus* locus genes, respectively. Changed activities of the modulons consisting of the inherently co-regulated genes enhanced understanding of the differential expressions of the EHEC virulence and survival genes in response to specific intestinal environments. Consequently, ICA can expand and enhance the current understating of the TRNs of EHEC, suggesting that ICA can provide broader insight into the TRNs of other pathogens from their transcriptome data.

## Data Availability Statement

The original contributions presented in this study are included in the article/Supplementary Material, further inquiries can be directed to the corresponding authors.

## Author Contributions

HI, J-HL, and SC: conceptualization, writing—original draft and review and editing. HI: methodology, validation, formal analysis, investigation, data curation, resources, and visualization. J-HL and SC: supervision, project administration, and funding acquisition. All authors contributed to the article and approved the submitted version.

## Conflict of Interest

The authors declare that the research was conducted in the absence of any commercial or financial relationships that could be construed as a potential conflict of interest.

## Publisher’s Note

All claims expressed in this article are solely those of the authors and do not necessarily represent those of their affiliated organizations, or those of the publisher, the editors and the reviewers. Any product that may be evaluated in this article, or claim that may be made by its manufacturer, is not guaranteed or endorsed by the publisher.

## Footnotes

1
https://www.ncbi.nlm.nih.gov/sra
