# Supplementary material for: Independent Component Analysis Identifies the Modulons Expanding the Transcriptional Regulatory Networks of Enterohemorrhagic Escherichia coli
Source: Front Microbiol. 2022 Jun 24;13:953404. doi: 10.3389/fmicb.2022.953404 (PMC9263587; doi:10.3389/fmicb.2022.953404)
Supplement: Supplementary file 5 [file Data_Sheet_5.DOCX]

**Supplementary Materials**

**Independent component analysis identifies the modulons expanding the transcriptional regulatory networks of enterohemorrhagic *Escherichia coli***

**Hanhyeok Im^1,2^, Ju-Hoon Lee^2,*^, and Sang Ho Choi^1,2,*^**

^1^ National Research Laboratory of Molecular Microbiology and Toxicology, Department of Agricultural Biotechnology, Seoul National University, Seoul 08826, Republic of Korea,

^2^ Deparment of Food and Animal Biotechnology, Deparmtent of Agricultural Biotechnology, Center for Food and Bioconvergence, Seoul National University, Seoul 08826, Republic of Korea

**^*^Correspondence:**

Dr. Ju-Hoon Lee / juhlee@snu.ac.kr

Dr. Sang Ho Choi / choish@snu.ac.kr

**Supplementary Table 1. Bacterial strains and plasmids used in this study**

| Strain or plasmid | Relevant characteristics^a^ | Reference or source |
| --- | --- | --- |
| Bacterial strains  *E. coli* | | |
| DH5α | *supE44 ΔlacU169* (*Φ80 lacZ Δ*M15) *hsdR17 recA1 endA1 gyrA96 thi-1 relAI* | Laboratory collection |
| EDL933 | Wild-type; clinical isolate; virulence | Laboratory collection |
| HH101 | EDL933 with Δ*ler* | This study |
| Plasmids | | |
| pCas | *repA101*(Ts) P*_cas_*-*cas9* P*_araB_*-*Red lacI^q^* P*_trc_-sgRNA-pMB1*; Km^r^ | (Jiang et al., 2015) |
| pTargetF | *pMB1* *aadA* *sgRNA-cadA*; Spc^r^ | (Jiang et al., 2015) |
| pTargetF-ler | pTargetF with *sgRNA-ler*; Spc^r^ | This study |

^a^Km^r^, kanamycin-resistant; Spc^r^, spectinomycin-resistant.**Supplementary Table 2. Oligonucleotides used in this study**

| Oligonucleotide | Oligonucleotide sequence (5’ →3’)^a^ | Use |
| --- | --- | --- |
| For mutant construction | | |
| LER-F1-F | AAAACATTTGCGGCTTCTTT | Deletion of *ler* ORF |
| LER-F1-R | CTACAGCAGGAAGCAGAAGCACTGTTGAATGGAATGAAGAAAGAAGATTT |  |
| LER-F2-F | ATTCAACAGTGCTTCTGCTTCCTGCTGTAGAACTGCAATTTGCTCTATAA |  |
| LER-F2-R | CAGGAAGGACCAACAATTAATCA |  |
| N20-LER-F | TTCTTCATTGGTTTTAGAGCTAGAAATAGC | Replacement of N_20_ of pTargetF |
| N20-LER-R | GGGCAGACCTACTAGTATTATACCTAGGAC |  |
| For qRT-PCR | | |
| GAPDH-qRT-F | AGGTCTGATGACCACCGTTC | Quantification of the *gapA* expression |
| GAPDH-qRT-F | AACGGTCAGGTCAACTACGG |  |
| Z0395-qRT-F | AAAGCCAGTCTCCTTCAACTC | Quantification of the Z0395 gene expression |
| Z0395-qRT-R | CTCGACAACACATCCTCTTCTT |  |
| STX2A-qRT-F | GAACGTTCCGGAATGCAAA | Quantification of the *stx2a* expression |
| STX2A-qRT-R | CCATTAACGCCAGATATGATGA |  |
| THIB-qRT-F | CGAAGGCGAAGTAGCCATAA | Quantification of the *thiB* expression |
| THIB-qRT-R | GTTAGACGCCGCCAGTAAA |  |
| THIC-qRT-F | CCGACGTGAAGTGGTCATAG | Quantification of the *thiC* expression |
| THIC-qRT-R | GCAATATGACCGAGGAGTTAGAG |  |
| CUSC-qRT-F | CGCTTAAAGAACATGAGCGAAG | Quantification of the *cusC* expression |
| CUSC-qRT-R | TAGCTTTGCGCGACATTA |  |

^a^Regions of oligonucleotides not complementary to the corresponding genes are underlined.


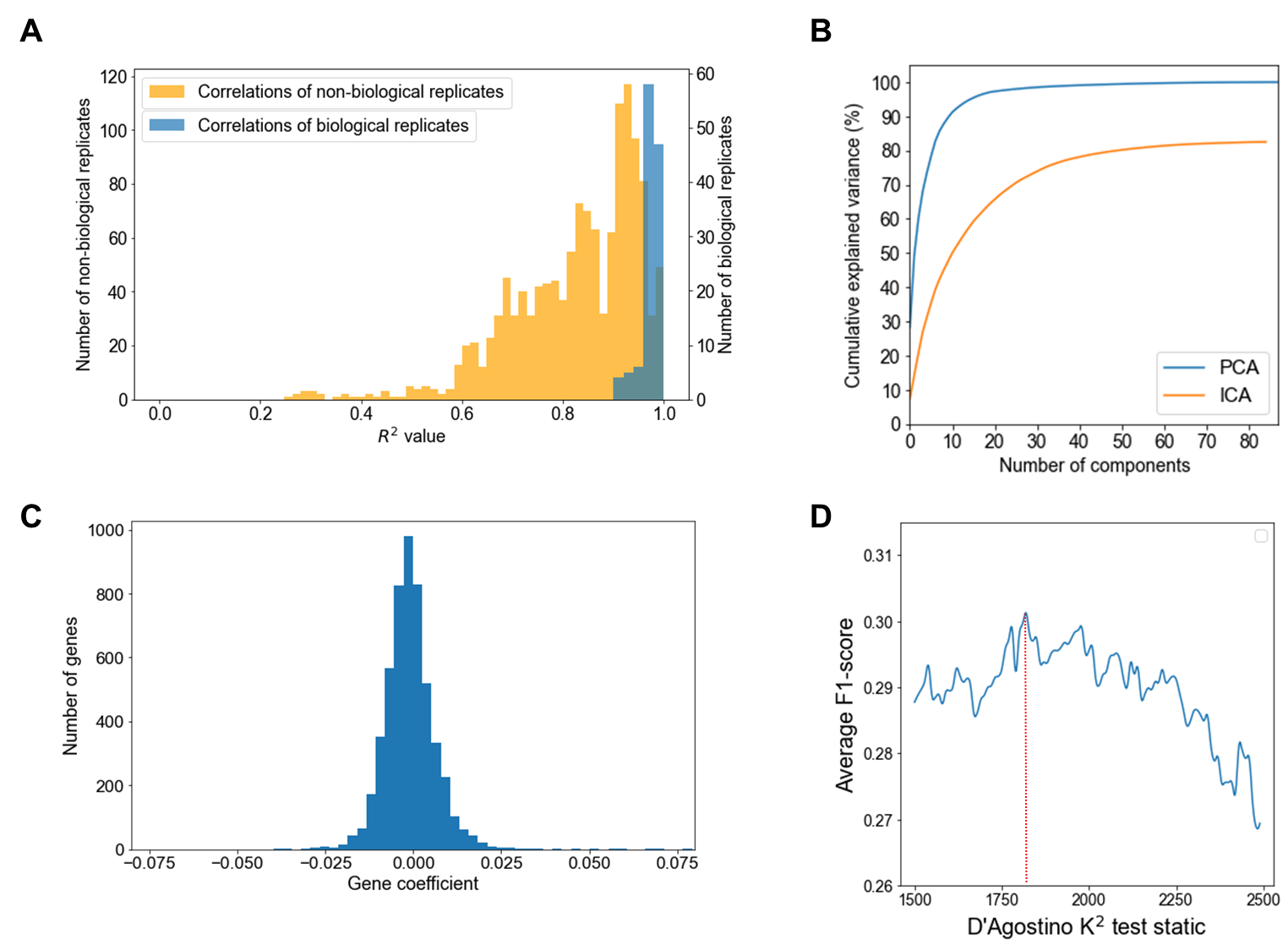


**Supplementary Figure 1.** Summary of the data processing. (A) Histogram of the correlation values between the biological replicates or non-biological replicates of the transcriptome data of EHEC. The correlations between the replicates were calculated using *R*^2^ values. Blue bars represent the correlations between the biological replicates, and the orange bars represent the correlations between non-biological replicates. (B) Cumulative explained variance (CEV) for the 88 transcriptome data of EHEC calculated by using the principal component analysis (PCA) (blue) and the independent component analysis (ICA) (orange). The independent gene components identified by using the ICA explained 83% of the total expression variance of the 88 transcriptome data. (C) Histogram of the gene coefficients in an independent gene component. The gene coefficients in any independent gene component display the histogram with a similar distribution. Most of the gene coefficients are near zero. (D) Average F1 scores calculated under the varied D’Agostino K^2^ statistic cutoff ranging from 1,500 to 2,500 with an increment 10. Red dotted line indicates the optimal cutoff value, 1800.

**
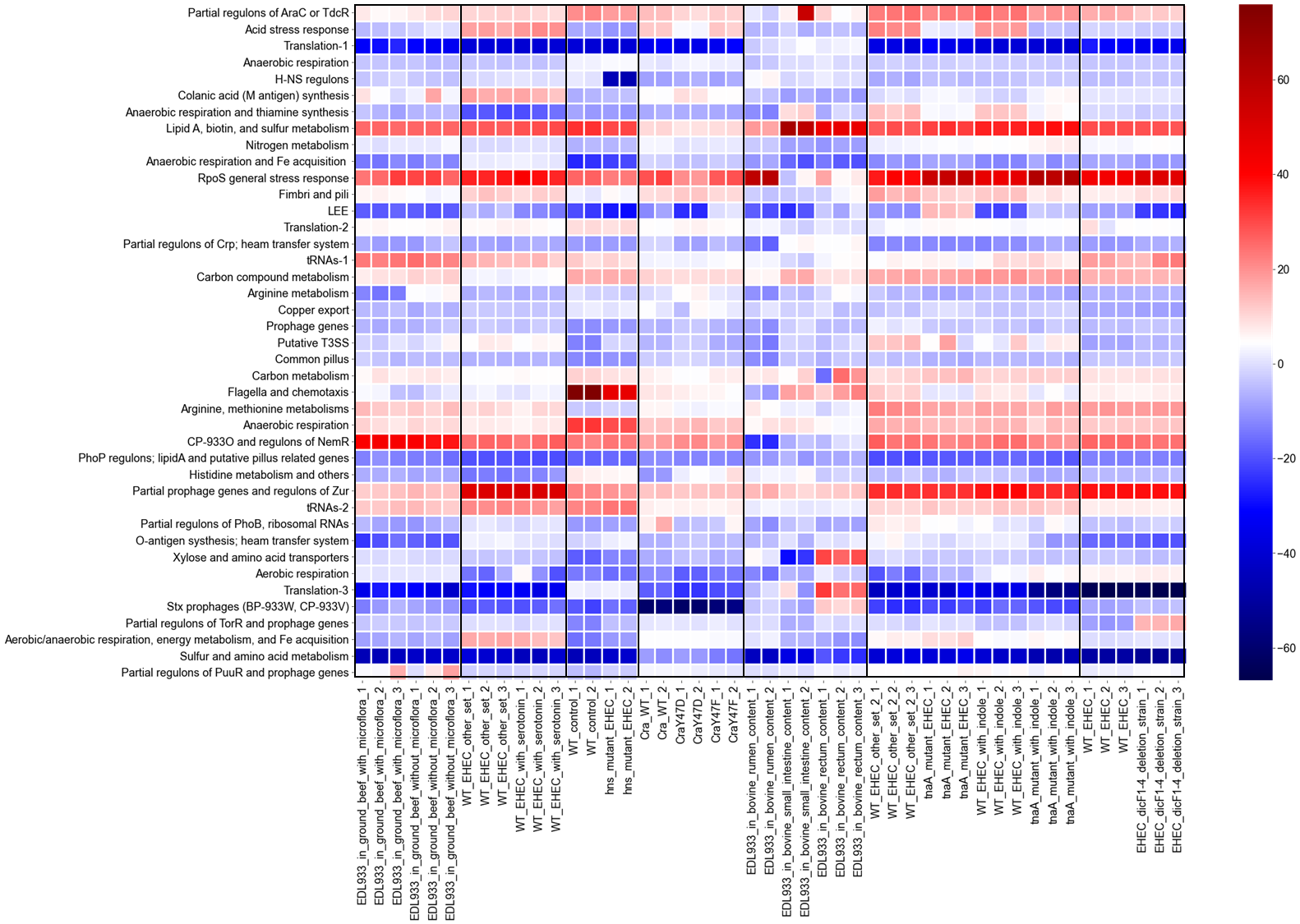
**

**Supplementary Figure 2.** Heatmap for the changed activities of the modulons obtained from the transcriptome data of EHEC under different experimental conditions. Only the modulons with known related TFs or biological functions were shown. The numbers on the labels indicate a distinct single biological replicate. Detailed experimental conditions of the transcriptome data can be found at Data Set S1. Detailed activities of the modulons of the transcriptome data can be found at Data Set S4. Red and blue represent the high and low activity of the modulon, respectively.
